# Mucoepidermoid Carcinoma of the Tongue Base Mimicking an Ectopic Thyroid

**DOI:** 10.1155/2013/925630

**Published:** 2013-02-07

**Authors:** Salvatore Martellucci, Giulio Pagliuca, Marco de Vincentiis, Chiara Rosato, Ettore Scaini, Camilla Gallipoli, Andrea Gallo

**Affiliations:** ^1^ENT Section, Department of Surgical Sciences, University of Rome “La Sapienza”, 00192 Rome, Italy; ^2^ENT Section, Department of Sense Organs, University of Rome “La Sapienza”, 00192 Rome, Italy

## Abstract

A 69-year-old woman with mucoepidermoid carcinoma (MEC) of the tongue base came under our observation complaining of repeated episodes of haemoptysis. Mucoepidermoid carcinoma of the tongue base gives rise to a rather vague and aspecific symptomatology. Early symptoms include foreign body sensation in the oral cavity, undefined paraesthesia, and sialorrhoea. With the progression of disease, dysphagia, otalgia, and painful swallowing are usually referred. We report a case of mucoepidermoid carcinoma of the tongue base mimicking an ectopic thyroid.

## 1. Introduction

Mucoepidermoid carcinoma (MEC) is a common malignant salivary gland neoplasm presumed to arise from the reserve cells of salivary gland ducts. This malignancy originates in both major and minor salivary glands, although it generally occurs in the parotid gland (89.6%), followed by submandibular gland (8.4%). Intraorally, it shows a strong predilection for the palate [1–3]. MEC was rarely reported in the tongue base [4, 5]. In this site, a differential diagnosis must be made not only with other malignancies but also with congenital and benign lesions such as ectopic tissues of thyroidal, epidermal, dermal, venous, and lymphatic origins [6–9]. We present a case of MEC of the tongue base showing the gross appearance of an ectopic thyroid and discuss its clinical presentation.

## 2. Case Report

**Figure 1:**
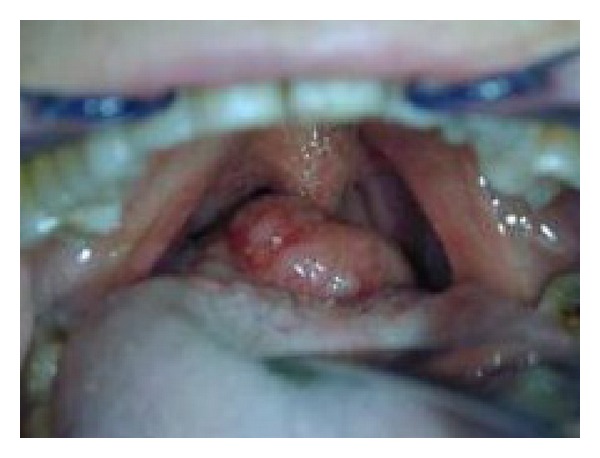
Photograph demonstrating the tumor on the base of the tongue.

A 69-year-old woman came under our observation following episodes of emophtoe. During the previous 5 months, the patient experienced sialorrhoea and worsening dysphagia. Clinical examination revealed the presence of a large, nodular formation with a smooth surface, richly vascularized, implanted in the midline of the tongue base (Figure 1). The macroscopic characteristics of the lesion suggested an ectopic thyroid diagnosis. CT imaging revealed a solid formation in the right area of the tongue base measuring 30 mm in diameter which occupied most of the oropharynx. No locoregional adenomegalies were detected. A thyroid scintigraphy and an ultrasound examination were carried out in order to determine whether the thyroid was in its normal location or in an ectopic one. No thyroid tissue was described at the tongue base, and the thyroid gland was described to be in its normal position. A selective arteriography of the external carotid arteries revealed that the mass at the tongue base was supplied by a rich vascular network deriving from the terminal branches of the tongue's artery bilaterally and by the maxillary artery from the left side. Selective embolization of the branches feeding the tumor was carried out with the aim of reducing haemorrhages and minimizing the risks of bleeding during a bioptic procedure. The histological examination showed the presence of a low-grade mucoepidermoid carcinoma originating from the minor salivary glands of the tongue base. The patient, once informed of the diagnosis and of the surgical treatment required to remove the neoplasm, refused to undergo any kind of therapy and was therefore discharged after being told about the risks she would run into in case she did not receive treatment.

## 3. Discussion

The base of the tongue may be the site of several benign and malignant tumors [6–8]. Usually, the clinical features of the tumor support a differential diagnosis, although the definitive diagnosis is reached after pathologic examination. The observation of a midline, not ulcerated, nodular mass with a rich vascular supply in the base of the tongue in a patient with a recent history of sialorrhoea and worsening dysphagia can suggest the presence of an ectopic thyroid [10, 11]. The tongue base is the most common localization of ectopic thyroidal tissue. Ectopic thyroid usually presents itself as a solid, pink, well-defined mass, covered with normal mucosa. The surface of the lesion is usually smooth, and a rich vascular supply can be seen. Scintigraphic and radiological examinations are necessary to confirm the diagnosis [10–12]. In this case, despite the suggestive macroscopic features, ultrasound examination revealed that the thyroid gland was in its normal position, and technetium scanning did not confirm the presence of thyroidal tissue at the base of tongue. The biopsy revealed the presence of low-grade MEC. 

MEC is a rarely reported clinical entity with no distinctive cytological characteristic. The tumor is composed of epidermoid and mucin-producing cells, which take origin from the duct epithelial lining. The epidermoid cells proliferate in sheets or islands, and keratinizing may occur. When the epidermoid constituent predominates, the histological appearance of the tumor may closely resemble that of squamous cell carcinoma, and it is thus classified as a high-grade MEC tumor. Conversely, the presence of mucin-producing cells within a predominately cystic architecture is regarded as low-grade MEC tumors [13]. Neck lymph node metastases are frequent, but their incidence, as well as the local aggressiveness of the tumor, is related to the degree of differentiation [14].

The progression of the tumor growth may cause serious hemorrhages, often making it necessary to tie the external carotid artery or to perform selective embolization. The histological characteristics and the natural tendency of this type of tumor to infiltrate surrounding tissue and cause lymph node metastases highlight the importance of a radical surgical approach, often combined with a neck dissection [15]. After surgery, radiotherapy may reduce the risk of locoregional recurrence in cases of advanced tumor with positive resection margins or with high-grade malignancy [15].
